# Old and New Concepts in Ubiquitin and NEDD8 Recognition

**DOI:** 10.3390/biom10040566

**Published:** 2020-04-07

**Authors:** Elena Santonico

**Affiliations:** Department of Biology, University of Rome Tor Vergata, Via della ricerca scientifica, 00133 Rome, Italy; Elena.Santonico@uniroma2.it

**Keywords:** ubiquitin, NEDD8, CUBAN, CoCUN, ubiquitin-binding domains, RNA metabolism

## Abstract

Post-translational modifications by ubiquitin and ubiquitin-like proteins (Ubls) have known roles in a myriad of cellular processes. Ubiquitin- and Ubl-binding domains transmit the information conferred by these post-translational modifications by recognizing functional surfaces and, when present, different chain structures. Numerous domains binding to ubiquitin have been characterized and their structures solved. Analogously, motifs selectively interacting with SUMO (small ubiquitin-like modifier) have been identified in several proteins and their role in SUMO-dependent processes investigated. On the other hand, proteins that specifically recognize other Ubl modifications are known only in a few cases. The high sequence identity between NEDD8 and ubiquitin has made the identification of specific NEDD8-binding domains further complicated due to the promiscuity in the recognition by several ubiquitin-binding domains. Two evolutionarily related domains, called CUBAN (cullin-binding domain associating with NEDD8) and CoCUN (cousin of CUBAN), have been recently described. The CUBAN binds monomeric NEDD8 and neddylated cullins, but it also interacts with di-ubiquitin chains. Conversely, the CoCUN domain only binds ubiquitin. CUBAN and CoCUN provide an intriguing example of how nature solved the issue of promiscuity versus selectivity in the recognition of these two highly related molecules. The structural information available to date suggests that the ancestor of CUBAN and CoCUN was a three-helix bundle domain that diversified in KHNYN (KH and NYN domain-containing) and N4BP1 (NEDD4-binding protein-1) by acquiring different features. Indeed, these domains diverged towards two recognition modes, that recall respectively the electrostatic interaction utilized by the E3-ligase RBX1/2 in the interaction with NEDD8, and the hydrophobic features described in the recognition of ubiquitin by CUE (coupling ubiquitin conjugation to ER degradation) domains. Intriguingly, CUBAN and CoCUN domains are only found in KHNYN and N4BP1, respectively, both proteins belonging to the PRORP family whose members are characterized by the combination of protein modules involved in RNA metabolism with domains mediating ubiquitin/NEDD8 recognition. This review recapitulates the current knowledge and recent findings of CUBAN and CoCUN domains and the proteins containing them.

## 1. Ubiquitination and Ubiquitin-Binding Domains: A General Overview 

Among all the known post-translational modifications, the covalent addition of ubiquitin and ubiquitin-like proteins represents the unique example in which the chemical group modifying the substrate is a protein [1]. The heterogeneity of substrates that are modified by the covalent addition of ubiquitin extends well beyond the limited number of substrates known to be modified by ubiquitin-like proteins (Ubls). Accordingly, the two major pathways of selective protein degradation in eukaryotic cells, which are the ubiquitin–proteasome pathway and lysosomal proteolysis, use ubiquitin as a tag to target cytosolic and nuclear proteins for degradation. On the other hand, the post-translational modification by Ubls affects almost all the biological processes and represents a key step for the correct functioning of essential molecular complexes.

**Figure 1:**
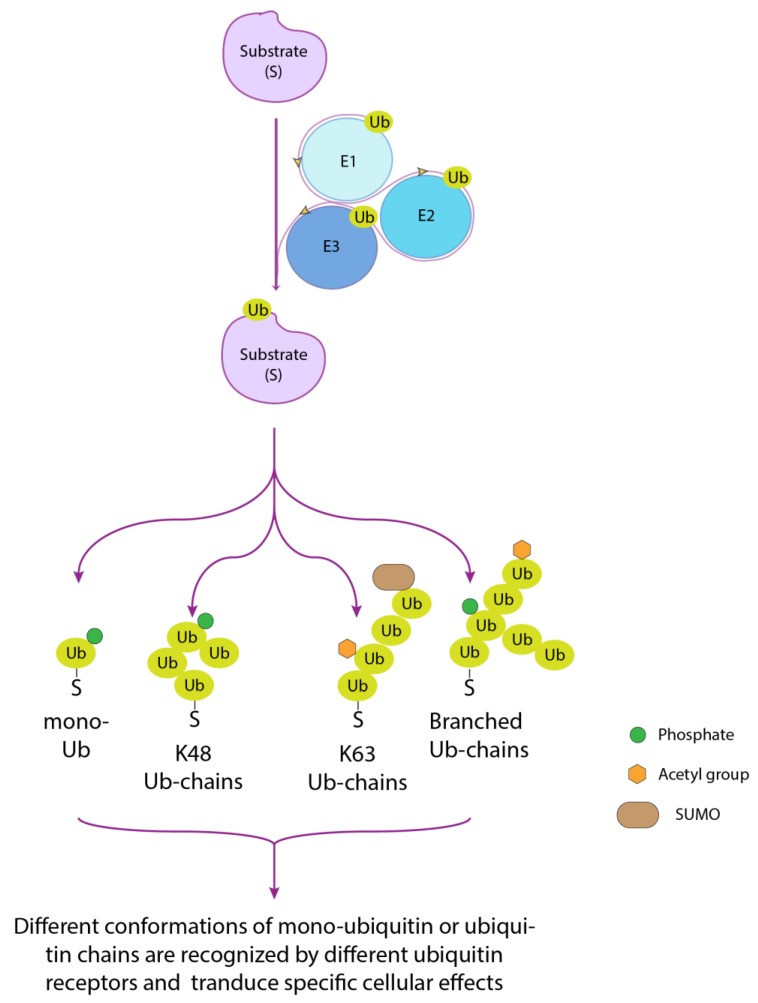
An enzymatic cascade involving an activating enzyme (E1), a conjugating enzyme (E2) and a ligase (E3) carries out protein substrate ubiquitination. The ubiquitin moiety conjugated to a substrate can be targeted for additional ubiquitination cycles, potentially involving all seven ubiquitin lysines (K6, K11, K27, K29, K33, K48 and K63) and the first methionine (M1). Different chain topologies are generated according to the specific branching. The conjugated ubiquitin moieties can be further modified by different post-translational modifications, such as phosphorylation, acetylation and sumoylation. Different polyubiquitin chains, as well as the presence of other post-translational modifications, determine differences in the conformations of the protein chains and therefore have specific effects on the protein to which they are attached. The association of protein post-translational modifications (PTMs) shown on the diagram is to be considered only as an example.

Ubiquitin is a 76 amino acid residues protein arranged into a β-grasp fold, a structural motif widely distributed in proteins of both eukaryotic and prokaryotic origin [2], that is characterized by a β-sheet with five antiparallel β-strands and a helical segment. The ubiquitinating pathway requires the coordinated activity of three enzymes, arranged in a cascade order, promoting the activation (E1), conjugation (E2) and recruitment of the substrate (E3) that is being ubiquitinated (Figure 1) [3]. The bond that is formed, called isopeptide, involves the α–carboxyl group of the terminal glycine (Gly) residue of Ub and the ε-amino group of an internal lysine (Lys) residue of the substrate. Albeit more rarely, cysteine, serine, threonine and the α-amino group of N-terminal methionine residue can also function as ubiquitination sites [4,5,6] The reversibility of this post-translational modification is ensured by deubiquitinating enzymes (DUBs) that cut the isopeptide bond, counteract substrate degradation and contribute to restoring the free ubiquitin pool. Another peculiarity of the ubiquitin system is that the first conjugated ubiquitin moiety, like any protein, constitutes the substrate for further ubiquitination reactions. Progressive cycles of modification following the first ubiquitination event lead to the formation of ubiquitin chains on the substrate protein. All seven ubiquitin lysines (K6, K11, K27, K29, K33, K48 and K63), as well as the first methionine (Met1), are potential ubiquitination sites [7], thus resulting in different types and hence chain topologies that will be generated according to the specific branching (Figure 1). Overall, the signals that can be potentially transmitted by a single protein are considerably diversified. For example, K48- and K63-linked ubiquitin chains are mainly involved in proteasome-mediated protein degradation and cell signaling, respectively [8]. Besides, mixed or branched chains can be generated, depending on whether different internal lysines are modified or different linkages within the same chain are generated [9,10,11]. To further enrich this panorama, conjugated ubiquitin molecules can be subjected to other post-translational modifications such as phosphorylation, acetylation, neddylation and sumoylation, thus adding a second layer of signaling (Figure 1) [12,13]. Based on this premise, the rigid conservation of the ubiquitin sequence along with the evolution, with only three amino acid substitutions differentiating the yeast and human ubiquitin proteins, can be interpreted as a necessary prerequisite. The base of such versatility, in fact, primarily lies in (1) the extreme variability with which individual units of ubiquitin can be combined, thus generating three-dimensional structures of the most diversified, and (2) the set of ubiquitin modifications within these structures. 

The recognition of monomeric ubiquitin and ubiquitin chains by a set of proteins, commonly called "ubiquitin receptors", is mediated by a plethora of different ubiquitin-binding domains (UBDs) [14,15,16]. As the binding affinity for monomeric ubiquitin is in the low micromolar range (with a Kd usually between 30 and 300 μM) [17], most ubiquitin receptors contain multiple domains arranged in tandem, each capable of recognizing ubiquitin and eventually the specific linkage between two ubiquitin moieties [15,16,17,18,19]. For example, tandem UIM (ubiquitin interacting motif), repeated up to four times, can be found in Eps15, epsins and other protein adaptors [20,21]. UBA (ubiquitin-associated) and A20 ZF (A20 Zn-finger) domains can be also found in multiple copies [22,23]. Alternatively, combinations of different families of ubiquitin-binding domains can be arranged in the same ubiquitin receptor, such as the VHS (Vps27p, Hrs and STAM)–UIM couple in STAM1/2 (signal transducing adapter molecule 1/2) proteins and HGS (hepatocyte growth factor-regulated tyrosine kinase substrate) [24,25] and UBAN (Ub-binding domain in ABIN proteins and NEMO)–ZF in NEMO (NF-kappaB essential modulator) [26]. This quite typical arrangement of UBDs in the same polypeptide chain, together with compartmentalization and oligomerization of ubiquitin receptors, improve the system avidity, thus allowing achieving a binding affinity for ubiquitin chains that is functional to the ubiquitin-mediated signal transduction [17]. On the other hand, mono-ubiquitination is also a physiological regulatory signal required in a variety of basic cellular processes, including endocytosis, histone remodelling, and viral budding [27]. The transduction of this signal usually requires intra-molecular interactions involving the recognition of the conjugated ubiquitin by a ubiquitin-binding domain in the same polypeptide chain [28].

To date, 25 different UBD families have been identified, many of which have spread in different proteins. In a limited number of cases, ubiquitin-binding domains represented by a unique member can be found [16,29,30]. Based on their structural fold, UBDs are categorized into three main groups. The largest group includes UBDs having an α-helical folding. Belong to this group are UBA, UIM, GAT (GGA and TOM), VHS, CUE (coupling ubiquitin conjugation to ER degradation), UBM (ubiquitin-binding motif), UBAN, UBW (ubiquitin-binding winged-helix domain), MyUb (myosin VI ubiquitin-binding domain), CARD (caspase recruitment domain), SOUBA (solenoid of overlapping UBAs), CUBAN (cullin-binding domain associating with NEDD8) and CoCUN (cousin of CUBAN). The second group is typified by zinc finger motifs in their structures and includes NZF (Npl4 Zinc Finger), A20_ZnF domains, UBP (ubiquitin-specific processing protease) and UBZ (ubiquitin-binding ZnF). The third group includes domains presenting β-sheets or α/β structures such as the Beta-Prp/WD40 (beta-propeller/WD40), UEV (UBC E2 variant), SH3 (Src homology 3), PRU (pleckstrin homology domain), GLUE (split-pleckstrin homology domain), Jab1/MPN (Jab1, Mpr1, Pad1 N-terminal), PFU (PLAA family ubiquitin binding) and Ubc (ubiquitin-conjugating)-related domains. Despite the high structural diversification between the different families of UBD, the vast majority of them recognize the binding surface constituted by residues Val8, Ile44 and Val70. As Ile44 is the center of the binding site, this functional surface is called the Ile44-centered hydrophobic patch. At least in some cases, however, Ile44 provides a minor contribution to the interaction [28]. In addition, alternative functional surfaces centered on residues Ile36, Asp58, Phe4 and the C-terminus (Gly-Gly76) can act as loci for binding to partners, leaving the hydrophobic patch available for other UBD-mediated interactions. The roles of these functional surfaces have been extensively reviewed elsewhere [15,17,19,29,31]. A relevant point emerging from the structural characterization of several UBD/ubiquitin interactions is that, whenever the UBDs recognize the most common hydrophobic patch, each domain shows a specific “footprint” on ubiquitin. In other words, residues peripheral to the hydrophobic patch act as determinants in the interaction of individual UBDs with the hydrophobic patch. It follows that domains sharing a similar structural arrangement can differ in the orientation used to engage the Ile44-patch [28].

A second level of diversification in the interaction surfaces of ubiquitin is given when the ubiquitin moiety is presented inside polyubiquitin chains. Therefore, the capability of different ubiquitin-binding domains to identify configurations characteristic of one type of internal ubiquitin linkage, but not another, allows each domain to transduce a signal that is specific for one or a few cellular pathways and cellular contexts. UIM motifs, for example, can recognize K48- or K63-linked ubiquitin chains depending on the ubiquitin receptor in which they are found. Indeed, the UIMs in the proteasomal subunit S5a/Rpn10, which select substrates for degradation, bind K48-linked ubiquitin chains [32,33,34]. Interestingly, they also interact with K63-linked tetraubiquitin in vitro, supporting the notion that they can also serve in the proteasomal recruitment of substrates conjugated to different polyubiquitin chains [35]. UIM motifs are also involved in nonproteolytic processes where they primarily recognize K63-linked ubiquitin chains. For example, they are frequently found in membrane-associated ubiquitin receptors that are required for the endocytosis of ligand-activated surface receptors and for the regulation of intracellular trafficking [36], as well as in proteins acting in the DNA damage response [37,38]. Among the best-known examples of UBDs involved in inflammation and immunity, UBAN-containing proteins, although highly conserved, exhibit different ubiquitin-binding characteristics resulting in diverse biological roles. The UBAN-containing proteins are NEMO, ABIN1-3 and optineurin. These domains preferentially bind M1-linked ubiquitin chains as well as K63-linked chains (although with lower affinity) but not K48-linked polyubiquitin chains [39,40,41]. The UBAN domains of NEMO (NF-κB essential modulator) and ABIN1 (A20-binding inhibitors of NF-κB) are essential for the regulation of inflammation, tumorigenesis and immunity [42,43,44]. Whilst the UBAN domain of NEMO is required for TNF-α (tumor necrosis factor)-induced NF-kB activation [39], the same domain in ABIN-1 binds to linearly ubiquitinated NEMO and A20 ubiquitin-editing enzyme and promotes deubiquitination and termination of NF-κB signaling [45]. Optineurin (OPTN) is a multifunctional adaptor protein that coordinates the homeostasis of many cellular processes, such as innate immune response, protein trafficking and organelle maintenance [46]. It has a master role as a selective autophagic receptor for the removal of a wide range of cargoes, such as intracellular pathogens and damaged mitochondria, and it also plays an important function in the innate immune response by negatively regulating NF-κB activity [47,48]. Notably, phosphorylation of UBAN domains plays a key role by enhancing binding to ubiquitin chains. Indeed, phosphorylation at S473 and S513 in the UBAN of OPTN, catalyzed by mitophagy-activated TBK1, increases its binding to M1-linked ubiquitin chains, further driving mitophagy [47,49,50,51]. The K63-linked polyubiquitination of TBK1, which is required for the kinase activation, is sensed and recruited by Golgi-localized OPTN that is, in turn, phosphorylated by the kinase, thus promoting further recruitment of the ubiquitinated cargo [52]. The phosphorylation of the ubiquitin-binding domain also increases the binding affinity of the UBA domain of SQSTM1 for polyubiquitin chains. SQSTM1 functions as a selective autophagy receptor for the degradation of ubiquitinated substrates. The UBA domain of SQSTM1 is phosphorylated by ULK1 and TBK1 kinases on Ser407 and Ser403, respectively. Phosphorylations occur at the early steps of autophagosome biogenesis and increase the binding affinity for ubiquitin chains, thus promoting selective autophagy of target proteins [53,54,55].

In conclusion, the convergence towards the recognition of a defined number of functional sites on the ubiquitin molecule by different families of ubiquitin-binding domains supports the notion that the coding sequence of the ubiquitin gene has been subjected to remarkable sequence conservation to strongly limit binding site alterations. At the same time, the selectivity of ubiquitin-binding domains for different ubiquitin chain types and the combination of different UBDs in the same polypeptide chain explain the distinct regulatory consequences observed in different signaling pathways. The multiplicity of the binding sites and the dynamic regulation of their binding properties further contribute to determining the specificity of ubiquitin-binding modules for ubiquitin.

## 2. Ubiquitin-Like Proteins

Ubiquitin-like (Ubl) proteins are known to regulate a strikingly diverse set of cellular processes, including proteolysis, autophagy, protein trafficking, inflammation, DNA repair, RNA splicing, transcription and translation [56,57,58,59]. They evolutionary expanded in eukaryotes, allowing a remarkable diversity in their interaction surfaces, in contrast with the high conservation of the ubiquitin sequence. They share with the founding member ubiquitin the typical β-grasp fold, similar chemistry of conjugation—based on an enzymatic cascade of three enzymes—and parallel regulatory processes [56,60]. Nine Ubl proteins have been identified (Table 1). As expected, conservation of the primary sequence was not a key requirement along with the evolution since, except for the neural precursor cell expressed, developmentally downregulated 8 (NEDD8) sharing a 60% identity with ubiquitin, other Ubls strongly diverged from the family founder [56,60]. In addition, a group of Ubls that does not exhibit covalent conjugation appeared and spread in the eukaryotic genome. They are called type II Ubls to distinguish them from type I Ubls, which are capable of covalent conjugation, and they occur as protein domains usually acting as protein–protein interaction sites. Ubls that exhibit global sequence conservation with ubiquitin bind their partners via a conserved Ile44-centered hydrophobic patch. Conversely, the contact sites involving Ubls that show a low level of sequence homology usually map in the surface called the α/β groove, a pocket formed by the α helix and β2 strand [28]. 

Unlike ubiquitin, which can be conjugated to virtually any cellular protein regardless of its function, type I Ubl proteins are characterized by a generally limited and functionally defined set of substrates. As an example, ATG12 is solely conjugated to ATG3 and ATG5 [68,69] and together with ATG8, the only Ubl that is conjugated to phospholipids [56] they are involved in autophagy. Structural analysis revealed that the scaffolding protein p62/SQSTM1 binds ATG8 by recognizing the groove and a conserved short N-terminal domain [70].

Small ubiquitin-like modifier (SUMO) has more diversified functions, and its cellular protein targets are involved in transcription, DNA repair and cellular stress response. SUMO family members, including up to four isoforms in the human genome, have similar structural and chemical characteristics but appear to be involved in different pathways. The SUMO conjugation pathway involves the SUMO activating enzymes SAE1/SAE2 and a unique E2 enzyme Ubc9 that conjugates SUMO proteins onto receptor lysines of the substrates. SUMO E3 ligases promote the last step while SUMO proteases—called ubiquitin-like protein-specific proteases in budding yeast (Ulps) or sentrin-specific proteases in mammals (SENPs)—disassemble the covalent modification [71,72]. Like ubiquitin, SUMO proteins can form polymeric chains, which are primarily disassembled by Ulp2p family proteases. A single lysine residue in vertebrates (K11) acts as an internal conjugation site, while three lysines (K11, K15 and K19) can be sumoylated in yeast [73].

SUMO proteins are not well conserved at the hydrophobic patch. SUMO-interacting motifs (SIMs or SBMs) have been described that interact with SUMO or SUMO conjugates, thereby regulating substrate functions. The first characterization of peptides that bind SUMO identified the consensus sequence [I/V/L]-K-X-[D/E], where X is any amino acid [66,74]. The cluster of aliphatic residues can be preceded or followed by three to four negatively charged amino acids [73,75]. Compared to ubiquitin-binding domains, SUMO binding peptides are smaller and display higher binding affinities, usually in the range of 1–10 Kd. The structural characterization of SUMO in complex with SIMs showed that SUMO binding peptides adopt an extended conformation that is inserted into the α/β groove of the Ubl, potentially in the two opposite orientations [76]. Lastly, an important set of SIM-containing proteins recognizes poly-SUMO conjugates. They are called SUMO-targeted ubiquitin ligases (STUbLs), and they ubiquitinate polysumoylated species, promoting their proteasomal degradation and thus linking SUMO modification to the ubiquitin/proteasome system [77,78].

### 2.1. The NEDD8 “Enigma”

Much less is known about binding domains that are specific for other Ubls. NEDD8, the closest relative of ubiquitin, is conjugated via the concerted action of the heterodimeric NEDD8 activating enzyme (APPBP1/UBA3, also called NAE1), the E2 UBC12 and the RING E3 enzyme RBX1/2 (RING-box 1/2) [56]. RBX1/2 is the ligase component of CRL (cullin-RING ligase) complexes, which are the largest known category of ubiquitin ligases. Nevertheless, to date, many noncullin substrates have been also described [11,79]. CRLs complexes regulate a plethora of cellular processes, including cell cycle, transcription, signal transduction and development [57,79]. Activation of CRLs occurs via the covalent attachment of NEDD8 to the cullin component, which is the scaffold of the complex. The C-terminus of cullins binds Rbx1/2, and it has been ascertained that the neddylation of cullins, promoted by Rbx1/2 itself, increases CRL ubiquitin ligase activity by allowing the RING E3 ligase to adopt the conformation that is functional to the enzymatic complex activity [80,81,82]. Interestingly, the structural characterization of the mechanism of NEDD8 ligation to CUL1 by Rbx1 revealed that NEDD8 directs the juxtaposition of the UBC12~NEDD8 active site and the CUL1 acceptor site [83]. This molecular mechanism involves a negative cluster Glu31–Glu32 in NEDD8 that contact RBX1’s Trp35, which is a key residue of the NEDD8 binding site. These interactions cannot be mimicked by ubiquitin, which brings the couple Gln–Asp in the same position. Notably, the swapping of residues 31 and 32 in ubiquitin with the corresponding amino acids in NEDD8 is sufficient to promote the ubiquitination of the C-terminal end of CUL1 by the E2 enzyme UBCH5, thus highlighting the crucial role of this pair in the discrimination between NEDD8 and ubiquitin by the CRL component RBX1 [83]. It follows that RBX1/2 is responsible for both the neddylation of cullins and the ubiquitination of any substrate recruited by the CRL complexes, once they have been activated.

Several reports show that NEDD8 forms chains in vitro and in vivo, although the function of polyneddylation is not well defined yet [10,78,84,85,86]. In addition, clear experimental evidence demonstrated that, upon proteotoxic stress, proteins are simultaneously modified by NEDD8 and ubiquitin mixed-chains. In particular, it has been suggested that NEDD8 functions as a less easily ubiquitinable substitute for ubiquitin, which caps the ubiquitin chain and prevents the excessive extension in those conditions in which the ubiquitin pool is depleted, first of all in proteotoxic stress conditions [87].

The NEDD8 pathway has a critical role in mediating the ubiquitination of numerous substrates involved in cell cycle progression, such as regulators of cell cycle and prosurvival signaling pathways (examples are cyclin E, p27 and the NF-κB inhibitor IκBα) [88]. Notably, several reports thoroughly reviewed in recent years have established that NEDD8 can be also conjugated by other E3-ligases (for example c-Cbl, MDM2, SMURF1/2), according to the notion that a neddylating enzyme with no E3-ligase activity for ubiquitin does not exist [11,79]. 

This "duplicity" is frequently associated with a certain ambiguity in the interpretation of the experimental evidence reporting the identity of neddylated substrates in vivo. Further enigmatic findings are that, under stress conditions, such as oxidative stress and proteasome inhibition, NEDD8 can be charged by the ubiquitin-activating enzyme UBE1 [87,89]. Similarly, under proteotoxic stress, the E3 ligase can target ribosomal proteins for neddylation, thus protecting the cell from ribosomal protein aggregation [9,11]. These examples of promiscuity, whether naturally occurring in specific cellular conditions or due to experimental strategies generating artifacts, still pose difficulties for an unambiguous characterization of the NEDD8 proteome.

The same ambiguity applies to NEDD8-binding domains that display cross-reactivity with ubiquitin, given the strong similarity of ubiquitin and NEDD8 in the Ile44-centered hydrophobic patch. The negative regulator of the NEDD8 pathway NUB1L (NEDD8 ultimate buster-1 long) and its splicing variant NUB1 were the first NEDD8-binding proteins to be described [61,62,90,91]. In both proteins, the consensus sequence A(X4)L(X10)L(X3)L at the C-terminal end is involved in the recognition of the Ubl. These motifs are embedded in the UBA domain within both variants and act by recruiting NEDD8 and its conjugates to the proteasome for degradation. Interestingly, the NUB1 and NUB1L UBA domains have been shown to bind to the small Ubl molecule FAT10 as well, most likely for their high sequence and structural similarities, resulting in the increased degradation of FAT10 and FAT10-modified cargos [92]. UBXN7, another NEDD8-binding protein, mediates the degradation of misfolded or damaged proteins that are ubiquitinated and directed to the proteasome. In particular, UBXN7 contacts and sequesters neddylated cullin2 via the UIM motif, downregulating the ligase activity of the CRL complex [63]. Notably, the replacement of NEDD8 with ubiquitin on cullin2 does not affect the interaction with UBXN7 [93], thus highlighting the promiscuity in the recognition by this UIM. Similarly, the UIM motif of hepatocyte growth factor regulated Tyr kinase substrate (HGS) promotes the recruitment of activated EGF receptor, which is ubiquitinated and neddylated in the cytoplasmic tail [94]. The nonproteasomal ubiquitin receptors hHR23a, UBQL1 and Ddi1 have been also shown to interact noncovalently with NEDD8 via their UBA domain and to deliver ubiquitinated and neddylated cargoes to the proteasome [95]. Lastly, a heterogeneous group of proteins (DNMT3b, TRIM40, NEDL1, NEDL2, DNA-PK, SMC1, AHR, BRAP2 and Rpt6) binds NEDD8 noncovalently, but the characterization of the recognition surface is still missing [11]. Interestingly, NEDD8-binding sites have been identified in several E3-ligases. In most cases, a ubiquitin-binding domain mediates the interaction with the Ubl, such as the motif interacting with ubiquitin (MIU) 2 domain of RNF168 that binds NEDD8 chains [96] or the UBA-like domains of TRIAD1 and HHARI, two members of the Ariadne family of RING-between-RING E3-ligases, that recruit neddylated CRL complexes [97]. Alternatively, sequences conforming to the consensus L(X7)R(X5)F(X)ALQ act as NEDD8 binding sites in the HECT domains of SMURF1 and SMURF2 E3-ligases. Despite the rapid growth of our understanding of the complexity within the cross-reactivity of NEDD8 with ubiquitin, we are still rarely able to establish whether neddylation is functionally distinct from ubiquitination. 

### 2.2. CUBAN and CoCUN: Similar but Different

As previously discussed, an important challenge in the field of ubiquitin-like signaling is to unravel the mechanisms underlying the specificity of ubiquitin-binding modules for Ubl molecules. The phage display approach has been recently applied to understand the determinants of ubiquitin and NEDD8 specificity recognition, leading to the identification of two novel ubiquitin and NEDD8 binding domains in the two evolutionary related proteins KHNYN (KH and NYN domain-containing) and N4BP1 (NEDD4-binding protein-1) [64,98,99]. Compared to previously characterized ubiquitin-binding domains, the selected regions show unique features. The domain identified in KHNYN, dubbed CUBAN for cullin-binding domain associating with NEDD8, spans residues 627–678. It interacts with monomeric NEDD8 (Kd = 24 ± 2 μM), but it also binds ubiquitin [64]. Moreover, while the interaction with monomeric ubiquitin is extremely weak, binding to di-ubiquitins—both linear and K48-linked type—is sufficiently strong to disrupt the association with free NEDD8 [93]. In addition to showing a clear preference for NEDD8 over monomeric ubiquitin, the CUBAN—both isolated and in the context of the full-length protein—promotes the association with neddylated cullins, thus clearly indicating a functional link with CRL complexes [93]. The interaction of CUBAN with neddylated cullins is independent from ubiquitination and only relies on the presence of the conjugated Ubl [64]. The CoCUN (cousin of CUBAN) domain, found in N4BP1 and spanning the C-terminal residues 847–896, shares 40% identity and 47% similarity with CUBAN [99]. The measured binding affinity of CoCUN for monomeric ubiquitin is 50 μM and, differently from the related CUBAN, it does not show any NEDD8-binding property. Intriguingly, the differences in the binding specificities of the two domains can be highlighted by analyzing the effect of the mutation Ala72Arg in the interaction of NEDD8 with both domains. Position 72 is known to be the main determinant for the discrimination between ubiquitin and the Ubl molecule by the E1 enzyme of the neddylation cascade. Accordingly, the presence of an arginine (Arg72) in ubiquitin, instead of the evolutionarily conserved alanine in NEDD8 (Ala72), prevents misactivation of ubiquitin by UBA3 as well as deconjugation by the deneddylating enzyme NEDP1 (NEDD8-specific protease 1) [100,101,102,103,104,105]. Interestingly, the “ubiquitinization” of the C-terminal tail of NEDD8 (A72R mutant) promotes the interaction with CoCUN and also with other UBDs that show low or no Ubl binding properties. On the contrary, this mutation affects the interaction with CUBAN, thus underpinning the specificity of NEDD8 recognition by this domain [64]. This evidence indicates that residue in position 72 modulates the binding preferences for ubiquitin and NEDD8 by those ubiquitin-binding domains that can potentially recognize both post-translational modifications. 

**Figure 2:**
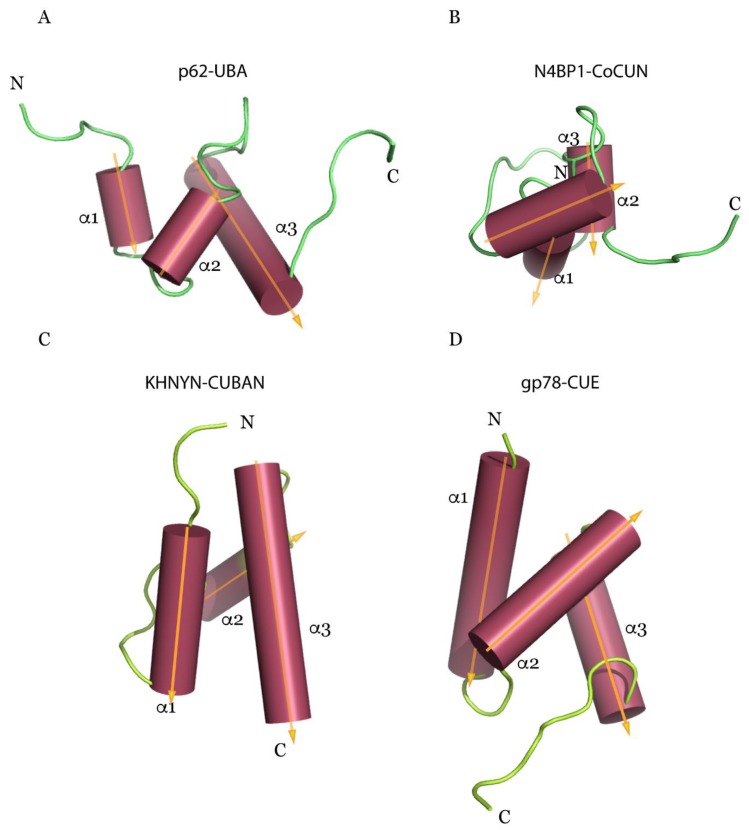
Ribbon representation of Ubiquitin-Associated (UBA) (p62, **A**), CUllin-Binding domain Associating with NEDD8 (CUBAN) (KH and NYN domain-containing (KHNYN), (**B**), Cousin of CUBAN (CoCUN) (NEDD4-binding protein-1 (N4BP1), (**C**) and Coupling Ubiquitin conjugation to ER degradation (CUE) (gp78, (**D**) domains. Arrows indicate the relative spatial distributions of the three helices (α1, α2 and α3). The central helix, located in front of the plane formed by helices 1 and 3 in CUE, UBA and CoCUN domains, is projected on the opposite side in CUBAN.

From a structural point of view, the investigation by NMR spectroscopy of CUBAN and CoCUN domains reported the presence of structural elements that are common to other UBDs belonging to the most populated category represented by a three-bundle helix, such as UBA and CUE [16,99]. Compared to the latter, a feature that is common to CUBAN and CoCUN domains is the unusually extended loop1, which is also typified by pronounced flexibility. Intriguingly, while the central helix of CUE, UBA and CoCUN is located in front of the plane formed by helices 1 and 3, in the CUBAN domain it is projected on the opposite side (Figure 2). 

**Figure 3:**
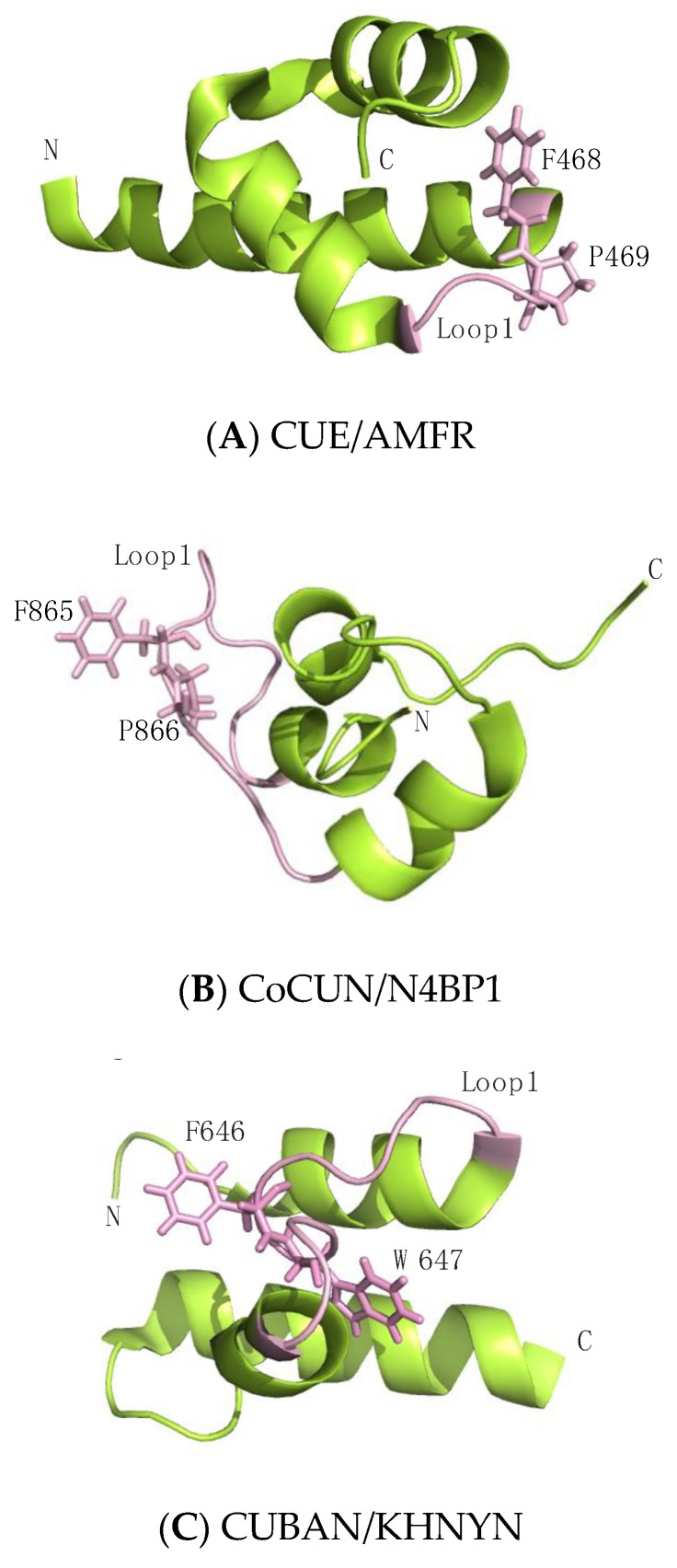
Cartoon representations of (**A**) the CUE domain of Autocrine Motility Factor Receptor (AMFR, PDB 4G3O), (**B**) the CoCUN domain of N4BP1 and (**C**) the CUBAN domain of KHNYN (PDB 2N5M). The FP/FW motifs are shown in sticks and the residues located in the loop1 are colored in light pink.

Interaction studies performed by NMR spectroscopy, associated with mutational analysis, revealed that (1) the Ile44-patch of ubiquitin represents the primary contact site of N4BP1 CoCUN domain and (2) an FP (_865_Phe-Pro_866_) motif, mapping in the loop1 of CoCUN domain, acts as the key element in the protein–protein interaction. Indeed, similarly to CUE/ubiquitin complexes, the FP motif recognizes the canonical hydrophobic patch of ubiquitin, and the Pro/Ala mutation disrupts the interaction between the two partners. In addition, the wild-type domain, but not the PA mutant, is ubiquitinated when transiently transfected in cells [99]. This feature is shared among CUE, UIM, MIU and A20 ZnF-containing proteins in which the ubiquitin-binding properties are coupled with the ubiquitination of the protein in which they are found, a process called coupled-monoubiquitination [17,106,107]. Differently from CUE domains where the FP motif is positioned at the C-terminal end of helix-1, in CoCUN the FP motif occupies a central position inside the extended loop1 and is also highly exposed to the solvent, at least in the absence of ubiquitin (Figure 3). Along with the high flexibility observed by NMR, this strongly suggests that the protein complex significantly affects the spatial position of the binding surface in CoCUN. The evidence that the CoCUN domain acquired functional characteristics that are common to the CUE domain family, despite showing key differences in the overall structure, provides a clear example of convergent evolution occurring between CoCUN and the CUE domains family.

Despite the significant homology, the CUBAN domain acts quite differently when compared to CoCUN. Indeed, the binding affinity of CUBAN/ubiquitin complex is undetectable by isothermal titration calorimetry (ITC), suggesting that the interaction with monomeric ubiquitin, whether it takes place, occurs in significantly distinct modalities. An FW motif (_646_Phe-Trp_647_), corresponding to the FP sequence characterized in CoCUN and also here positioned inside the extended loop1, is not involved in the recognition of NEDD8 nor in the ubiquitination of CUBAN. On the contrary, this amino acid couple points towards the core of the domain, and it appears to reduce the flexibility of loop1, thus performing a stabilizing function (Figure 3) [64,99]. From a structural point of view, the marked difference observed in the FP and FW motifs is the result of the different spatial distribution of the helix-2 in CUBAN compared to other three-helix bundle domains, as previously discussed (Figure 2). 

Therefore, despite the evolutionary relationship, the recognition mode of CUBAN and CoCUN for the respective ligands is mediated by structural elements that are strongly distinctive for each of the two domains. Accordingly, the molecular details of the CUBAN/NEDD8 complex reveal the electrostatic nature of this interaction, in contrast with the hydrophobic nature of most of the ubiquitin interactions involving the Ile44-centered patch, including the CoCUN/ubiquitin complex. CUBAN binds to residues in the second β-strand (Ile13 and Glu14) and the C-terminal end of helix α1 (31-Glu-Glu-Lys-Glu-34) of NEDD8. As expected, the mutations Glu31Gln and Glu32Asp, which substitute the NEDD8 sequence with the corresponding residues in ubiquitin, as well as the substitution with opposite charges (Glu31Lys and Glu32Lys) cause complete loss of binding [64]. From the CUBAN side, the binding interface is characterized by the positively charged residues His651, Arg652, Lys659 and Arg664 mapping in turn1, helix α2 and turn2 [64]. Interestingly, the molecular details of this interaction are partially reminiscent of the electrostatic interaction between RBX1 and the acidic residues Glu31 and Glu32 in NEDD8 [83]. Like RBX1, therefore, the CUBAN shows a dual-specificity towards ubiquitin and NEDD8 and the recognition of the same electrostatic surface on the Ubl molecule. 

Another interesting possibility is that the CUBAN domain could be involved in the signal transduction of mixed NEDD8–ubiquitin chains. Indeed, being able to interact with both ubiquitin and NEDD8, the CUBAN has the potential capability to discriminate among polyubiquitination, polyneddylation and mixed NEDD8–ubiquitin signals, thus providing a useful approach for the comprehension of the cross-talk between these two post-translational modifications.

In conclusion, the investigation carried out to date indicates that, starting from a common ancestor, the CUBAN and CoCUN domains diverged towards different recognition modes that recall the features observed, on the one hand, in the interaction of NEDD8 with the E3-ligase RBX1/2 and, on the other hand, in the recognition of ubiquitin by CUE domains. Future research will help in clarifying the molecular details of these novel protein complexes and their regulation in the context of the full-length proteins.

## 3. The PRORP Family Members

**Figure 4:**
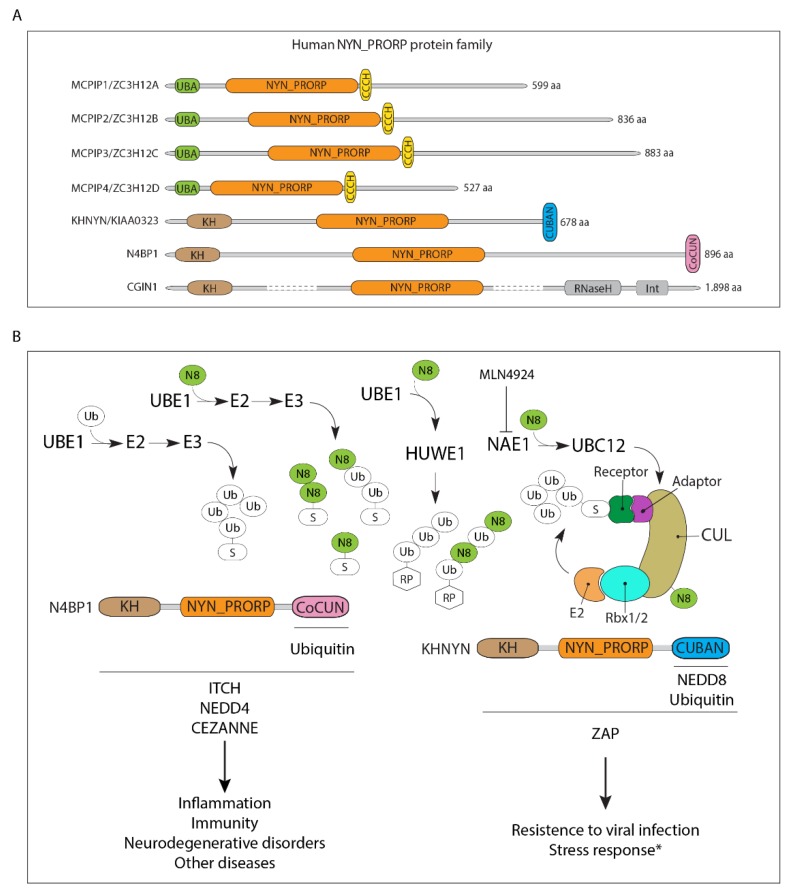
(**A**) Domain organization of human NYN_PRORP domain-containing proteins. (**B**) Protein–protein interactions and contextual network diagram of KHNYN and N4BP1 proteins. The ubiquitin E1 enzyme Ube1 mediates NEDD8 activation under diverse stress conditions. Modified neddylated proteins are simultaneously ubiquitinated. Upon proteotoxic stress the conjugation by the E3-ligase HUWE1 results in the modification of nuclear substrates, among them ribosomal proteins, with NEDD8, ubiquitin and hybrid NEDD8/ubiquitin chains. Cullin-RING ligases (CRLs) are functional multi-subunit complexes including substrate receptors, adaptors, cullin scaffolds and RING-box proteins. Domain organization and protein interactions of N4BP1 and KHNYN are shown together with the identified binding sites, where known. The biological processes in which these interactions are potentially involved are shown. (*) The involvement of KHNYN in the NEDD8-mediated stress response is only postulated. *Abbreviations*: UBE1, ubiquitin-activating enzyme; RP, ribosomal protein; HUWE1, HECT, UBA and WWE domain-containing E3 ubiquitin protein ligase 1; S, substrate; NAE1, NEDD8-activating enzyme E1; CUL, cullin; RBX1/2, RING-box 1/2; KH, hnRNP K homology; NYN_PRORP, KH and NYN domain-containing_PROteinaceous only RNase P; CoCUN, cousin of CUBAN; CUBAN, cullin-binding domain associating with NEDD8; ITCH, itchy E3 ubiquitin protein ligase; NEDD4, neural precursor cell expressed developmentally downregulated protein 4; CEZANNE, cellular zinc finger anti-NF-kappaB; ZAP, zinc finger antiviral protein.

The biological properties and cellular functions of KHNYN and N4BP1 are currently very poorly characterized, and much of the information available in the literature is indirect or deducible from the analysis of their domain organization. According to a bioinformatics approach, a link with processes related to RNA metabolism can ben deduced for both proteins. Their N-terminal end contains a hnRNP K-homology (KH) domain, which is a single stranded nucleic acid binding domain mediating the RNA target recognition, found in both eukaryotes and prokaryotes [108,109,110]. Following the KH, there is an NYN (NEDD4-BP1 and YacP nucleases) domain, belonging to the PIN-like (Pil-T amino-terminal) superfamily, identified for the first time in the ATPase Pil-T, which includes different structural groups of nuclease families. The analysis of known and predicted structures suggested that the NYN domains of these proteins are distinct from the typical NYN-like fold and should be considered rather members of the PROteinaceous-only Rnase P (PRORP) group, also including the KHNYN-related protein CGIN1 (NYNRIN), the monocyte chemotactic protein-induced protein 1 (MCPIP1, also known as regnase 1) and the related proteins MCPIP2, MCPIP3 and MCPIP4 [111] (Figure 4A). In all PRORP family members, the predicted RNA nuclease activity is combined with an RNA-binding domain. In particular, a hnRNP K homology (KH) domain is positioned upstream to the nuclease domain in CGIN1, KHNYN and N4BP1, while a CCCH domain (C-x8-C-x5-C-x3-H) maps downstream to the NYN domain in MCPIP1-4 proteins. Based on the current knowledge, MCPIP-1, which is the most characterized member of the PRORP family, acts as a negative regulator of the immune response by controlling the stability of the proinflammatory cytokines IL-6 and IL-12β, both actively transcribed following the stimulation of Toll-like receptors [112,113]. MCPIP1 also shows a protective role in antiviral defense by degrading viral RNAs [114], and it is a suppressor of miRNA biosynthesis by catalyzing the cleavage of the terminal loops of precursor miRNAs (pre-miRNAs) [115].

N4BP1 was initially characterized as a binding partner of the HECT-ligases NEDD4 and Itch [116]. Interestingly, N4BP1 inhibits Itch’s activity by out-competing Itch’s substrates [117] (Figure 4B). The protein localizes in the nucleoli where it is subjected to various post-translational modifications, such as sumoylation and ubiquitination [118]. Similarly to the other members of the PRORP family, a function in the inflammatory response is also profiled for KHNYN. Accordingly, it has been shown to act as a cofactor for the antiviral response mediated by the zinc finger antiviral protein (ZAP), which lacks enzymatic activity and recruits other cellular proteins to inhibit viral replication. It has been suggested that KHNYN could interact with ZAP and recognize the CpGs regions in the viral RNA genome, thus inhibiting its replication [119]. 

Alongside the RNA binding and processing functions, the PRORP family members interact in different manners with the ubiquitin system. MCPIP-1 contains a UBA domain at the N-terminus, but the presence of a similar domain in the other MCPIP members has not been experimentally validated [120,121,122]. The MCPIP proteins are also endowed with a deubiquitinating activity that targets proteins involved in the inflammatory response. To date, this function has been shown to require the UBA and the CCCH domains in MCPIP-1 [122]. N4BP1 also acts in the inflammatory response by interacting with the deubiquitinating enzyme CEZANNE, and it contains a C-terminal CoCUN domain mediating the interaction with ubiquitin [64,99,123]. Nevertheless, the CEZANNE-binding site has not been investigated yet. KHNYN contains a CUBAN domain that recognizes NEDD8 and ubiquitin and recruits CRL complexes through the interaction with recognizing specific ribonuclease complexes and mediating their degradation. In particular, whilst both proteins potentially target the RNA component via the KH domain and the putative RNase activity provided by the NYN domain, KHNYN could also act on the protein component through the CRL-mediated ubiquitination and proteasomal degradation. Nevertheless, the linkage with RNA metabolism as well as the importance of ubiquitin/NEDD8 binding of most PRORP family members in the regulation of inflammatory pathways has not been firmly established yet. We expect that future investigation will shed light on how all these biological properties are orchestrated.

To date, the functional role of CoCUN and CUBAN domains in the context of the respective full-length proteins has not been experimentally analyzed. KHNYN appears to be primarily a cytoplasmic protein, mostly aggregated to membrane compartments [124], that potentially shuttles between nucleus and cytoplasm in virtue of the presence of a nuclear localization signal [98]. KHNYN is also consistently ubiquitinated in cells, and such covalent modification is abrogated in the absence of the CUBAN domain, which therefore appears to be essential for the correct post-translational modification of the mature protein [64]. The CGIN1 protein, which is the last member of the PRORP family, is believed to have a retroviral origin. Notably, the CGIN1 gene resulted from a duplication of the KHNYN gene followed by the integration of a retroviral C-terminal segment containing the sequences coding for a putative RNase H domain and the integrase domain. The recombination eliminated the C-terminal region containing the CUBAN domain, which is uniquely present in KHNYN. The location of the CGIN1 gene, adjacent to KHNYN in the human genome, strongly supports this assumption [125]. To date, no link with ubiquitin has been investigated for CGIN1. Phylogenetic analysis shows that CGIN1 appeared in the branch of marsupials and eutherians, therefore after the monotreme split from the rest of mammals. On the contrary, KHNYN and N4BP1 orthologues are distributed in the three mammal lineages (monotremes, marsupials and placentals) [125]. This observation suggests that they are involved in biological processes that originated at early times during the evolution of mammals. 

## 4. Concluding Remarks

The current state of our knowledge in the field of ubiquitin and ubiquitin-like proteins highlights that, although each Ubl family covers selected activities and is conjugated to a restricted substrate panel, we frequently observe the intersection between distinct Ubl families. Ubiquitin itself can be modified by other Ubls, thus indicating that these modifications have significant degrees of crosstalk, which coexist with their peculiar distinguishing marks. The characterization of the two evolutionarily related domains CUBAN and CoCUN fully confirms this notion. Indeed, the structural analysis of CUBAN highlighted that two different partially overlapping binding sites are responsible for the interaction with ubiquitin and NEDD8 in the same domain, suggesting an intimate connection between the signal transduction of these posttranslational modifications. Given the evolutionary related origin of N4BP1 and KHNYN, we could assume that their ancestor was a ubiquitin-binding protein and that the gene duplication allowed KHNYN to acquire the capability to discriminate NEDD8 while shifting from mono- to polyubiquitin recognition. Moreover, the unique presence of CUBAN in the KHNYN protein suggests that it plays a role in a biological process that is mammal-specific.

Last but not least, several reports have demonstrated that in multiple cancers the neddylation pathway is overactivated and NEDD8-conjugated proteins are overexpressed, leading to the degradation of many tumor suppressor substrates of CRLs [126,127,128]. The discovery that the inhibition of protein neddylation could be an effective approach for cancer treatment paved the way to preclinical studies aimed to address toxicity and efficacy of several small-molecule inhibitors. The goal is to develop treatment protocols based on the combination of neddylation inhibitors with the anticancer therapy. MLN4924, developed by Millennium Pharmaceuticals, is a highly selective inhibitor of the catalytic subunit of NAE1. MLN4924 inactivates the neddylation cascade and exerts significant anticancer effects against solid tumors and hematological malignancies [126,129]. Indeed, treatment with MLN4924 induces the accumulation of cell-cycle-regulated CRLs substrates and proapoptotic proteins, leading to growth arrest, apoptotic cell death, senescence and autophagy in a cell-type-dependent manner [129,130]. An alternative approach could take advantage of peptides that target neddylated substrates and promote their proteasomal degradation. In the recent years, a number of E3-ligases have been reported to have both ubiquitylation and neddylation activity (reviewed in [11,79]). The dual specificity of these E3 enzymes, such as Mdm2, c-Cbl and SMURFs, lead to the identification of several noncullin substrates, as previously discussed [86,131,132,133]. The physiological meaning of these neddylation reactions is usually poorly defined, and the neddylated forms of noncullin substrates are hardly detectable under physiological condition. Nevertheless, neddylation of transcription factors such as p53 and E2F-1 has been shown to decrease their transcriptional activity [131,134] with or without affecting their degradation, thus highlighting the importance of a deeper understanding of NEDD8 conjugation. The identification of NUB1, NUB1L and their biding partner P97/VCP (valosin-containing protein, Cdc48 in yeast) as mediators of the proteasomal degradation of NEDD8 and NEDD8 conjugates [62,90,135] provides an additional approach for possible future therapeutic intervention. The NEDD8 binding properties of CUBAN could be also engineered in order to develop a molecular trap. Indeed, the knowledge of the binding surfaces involved in the specific recognition of NEDD8 by KHNYN can provide the source for designing peptide-based reagents to either block a macromolecular interface or exclude an individual component from a complex. This could allow, as for MLN4924, the development of reagents that lower the E3-ligase activity of specific CRL complexes, thus promoting the stabilization of substrates that induce cell-cycle arrest and apoptosis in cancer cells. Finally, dysfunctions of E3s of the NEDD4 family have been frequently identified in human cancers and other diseases [136,137,138,139]. Even though we need to further elucidate the relative contribution of the CoCUN domain to Itch E3-ligase inhibition by N4BP1, the capability of N4BP1 to interfere with the enzymatic activity of Itch, could provide a means to selectively block downregulation of Itch targets.

In conclusion, the recent expansion of our knowledge of ubiquitin and Ubl-binding domains opens up many new questions, only partially highlighted here. What is the functional role of ubiquitin/NEDD8 recognition in KHNYN and N4BP1? What are the underlying molecular mechanisms of specificity and cross-reactivity? What RNAs and/or ribonucleoprotein complexes are targeted by these proteins? How all this is related to the inflammatory response, and in what other biological processes do KHNYN and N4BP1 play a role? We expect that future research will help to answer these questions.

## Figures and Tables

**Table 1:** Known ubiquitin-like proteins and the protein domains that specifically recognize them.

Type-I UBL	Protein Name	Binding Domain	Reference
UBQ	Ubiquitin	UBDs	[15]
NEDD8	Neural precursor cell expressed, developmentally down-regulated 8	CUBAN, UIM, UBA	[61,62,63,64]
FAT10	Human Leukocyte Antigen F Locus Adjacent Transcript 10	UBA	[65]
ISG15	Interferon-stimulated gene 15	Specific ISG15-interacting motifs have not been identified	
SUMOs	Small ubiquitin-like modifiers	SIM (SUMO-interacting motif)	[66]
Atg8	Autophagy-related protein 8	AIM (ATG8-family interacting motif)	[67]
Atg12	Autophagy-related protein 12	Specific Atg12-interacting motifs have not been identified	
URM1	Ubiquitin-related modifier-1	Specific URM1-interacting motifs have not been identified	
UFM1	Ubiquitin-fold modifier 1	Specific UFM1-interacting motifs have not been identified	

Only noncovalent interactions are here reported. The enzymes that interact with the ubiquitin-like protein (Ubl) in the conjugation process are not included. FUB1 (Fan ubiquitin-like protein 1) and HUB1 (histone monoubiquitylation 1) proteins, despite showing a Ubl fold, have not been observed conjugated to substrates [51].
